# Investigation of the Effects of Calendula officinalis on Gingival Tissue in Streptozotocin-Induced Diabetic Rats

**DOI:** 10.7759/cureus.118178

**Published:** 2026-10-19

**Authors:** Ismail Tekbas, Betul Yalcin, Esra Bozgeyik, Metin Calisir

**Affiliations:** 1 Department of Periodontics, Faculty of Dentistry, Adıyaman University, Adıyaman, TUR; 2 Department of Histology and Embryology, Adıyaman University, Adıyaman, TUR; 3 Department of Medical Services and Techniques, Adıyaman University, Adıyaman, TUR; 4 Department of Periodontology, Faculty of Dentistry, Adıyaman University, Adıyaman, TUR

**Keywords:** calendula officinalis, cox-2, gingiva, il-1 beta, type 2 diabetes mellitus

## Abstract

Objective: This study aimed to evaluate the effects of *Calendula officinalis* administration on inflammatory changes in gingival tissue in a streptozotocin-induced experimental diabetes model.

Methods: Twenty-eight male Sprague-Dawley rats were randomly divided into four groups (n=7): control (C), *Calendula officinalis* (CO), diabetes mellitus (DM), and diabetes mellitus + *Calendula officinalis* (DM+CO). Diabetes was induced by intraperitoneal administration of streptozotocin (50 mg/kg), and *Calendula officinalis* extract was administered by oral gavage at 250 mg/kg for six weeks. Gingival cyclooxygenase‑2 (COX-2) and interleukin-1β (IL-1β) levels were determined by ELISA and normalized to total protein concentrations. Gingival tissues were also evaluated histopathologically using hematoxylin and eosin and Masson's trichrome staining.

Results: Gingival COX-2 and IL-1β levels were significantly higher in the DM group than in the control group (*p*=0.011 and *p*=0.017, respectively). In the DM+CO group, both COX-2 and IL-1β levels were significantly lower than those in the DM group (*p*=0.026 for both). Histopathological evaluation revealed epithelial and connective tissue irregularities in the DM group. In the DM+CO group, the epithelial-connective tissue junction appeared more regular and distinct, and connective tissue organization exhibited a more regular morphological appearance than in the DM group.

Conclusion: *Calendula officinalis* administration was associated with lower gingival COX-2 and IL-1β levels in STZ-induced diabetic rats. The accompanying histopathological observations suggest that its anti-inflammatory effects may also be reflected in gingival tissue morphology. Further studies incorporating quantitative histomorphometric analyses and broader inflammatory and oxidative stress markers are warranted.

## Introduction

Diabetes mellitus (DM) is a metabolic disease characterized by chronic hyperglycemia and causes numerous metabolic, immunological, and inflammatory alterations in the human body. Advanced glycation end products (AGEs), increased production of reactive oxygen species (ROS), mitochondrial dysfunction, and cellular stress responses resulting from hyperglycemia contribute to the persistence of chronic inflammation in diabetic tissues [1,2]. These biological alterations in diabetic tissues have been reported to increase the production of interleukins, tumor necrosis factors, chemokines, growth factors, prostaglandins, and various inflammatory mediators, thereby activating multiple cellular signaling pathways [3,4]. Nuclear factor kappa-B (NF-κB) [5], mitogen-activated protein kinase (MAPK) [6], Janus kinase/signal transducer and activator of transcription (JAK/STAT) [7], cyclooxygenase (COX) [8], and NLRP3 inflammasome [9] pathways are known to play important roles in DM-associated inflammatory processes. Activation of these pathways results in increased release of interleukin-1β (IL-1β), interleukin-6 (IL-6), interleukin-17 (IL-17), tumor necrosis factor-α (TNF-α), interferon-γ (IFN-γ), and various chemokines [10-13]. Suppression of antioxidant defense mechanisms and the concomitant increase in oxidative stress resulting from hyperglycemia contribute to the persistence of inflammation and progression of cellular damage [14]. Accordingly, the chronic inflammatory microenvironment and systemic inflammatory alterations induced by diabetes have been reported to affect various oral tissues, including periodontal tissues [15,16]. Increased oxidative stress, microvascular alterations, impaired immune cell function, and increased release of inflammatory mediators associated with hyperglycemia are thought to contribute to an exacerbated inflammatory response in periodontal tissues [17]. In addition, impaired collagen metabolism, delayed tissue healing, and increased inflammatory cell infiltration under diabetic conditions may adversely affect the homeostatic balance of periodontal tissues [18].

In recent years, phytotherapeutic agents with anti-inflammatory and antioxidant properties have attracted increasing interest in dentistry, particularly for their potential role in modulating gingival and periodontal inflammatory responses [19-21]. Among these agents, *Calendula officinalis* exhibits anti-inflammatory [22], antioxidant [23], antimicrobial [24], and tissue healing-promoting properties owing to its rich biological content of flavonoids, phenolic compounds, triterpenoids, and carotenoids. Experimental studies have reported that *Calendula officinalis* may exert beneficial effects on reducing oxidative stress, suppressing inflammatory mediators, and modulating various cellular signaling pathways. Furthermore, some studies have demonstrated beneficial effects of *Calendula officinalis* on wound healing, collagen organization, inflammatory cell infiltration, and tissue regeneration [25]. Considering the extensive vascularization of gingival tissues and their susceptibility to inflammatory stimuli, *Calendula officinalis* may have potential beneficial effects on the modulation of gingival inflammatory processes.

Nevertheless, studies investigating the effects of *Calendula officinalis* on gingival inflammatory processes associated with diabetic conditions remain limited. Therefore, the aim of this study was to evaluate the effects of *Calendula officinalis* administration on gingival inflammatory responses in rats with streptozotocin (STZ)-induced diabetes, primarily by assessing gingival cyclooxygenase-2 (COX-2) and IL-1β levels, together with supportive qualitative histopathological observations.

## Materials and methods

Study design and ethical approval

This study was an experimental laboratory investigation conducted on gingival tissue samples obtained from rats with STZ-induced experimental diabetes. The study protocol was reviewed and approved by the Adıyaman University Local Ethics Committee for Animal Experiments (HADYEK) (Decision No: 11, Protocol No: 2026/12). All experimental procedures were performed in accordance with international ethical principles for animal research.

Experimental animal model

A total of 28 male Sprague-Dawley rats, aged 8-10 weeks and weighing 200-250 g, were used in the original experimental study. The animals were housed under standard laboratory conditions (21 ± 1°C, 12-h light/12-h dark cycle) and provided ad libitum access to standard pellet feed and water.

The animals were randomly allocated into four experimental groups (n = 7 per group) using a simple lottery-based randomization procedure: Control group (C); Calendula officinalis group (CO); Diabetes mellitus group (DM); Diabetes + Calendula officinalis group (DM+CO).

To minimize potential assessment bias, the researchers responsible for biochemical analyses and histopathological evaluations were blinded to the experimental group assignments. Group allocation information was withheld from these researchers throughout the evaluation process. Experimental diabetes was induced by intraperitoneal administration of streptozotocin (50 mg/kg) prepared in 0.1 mol/L citrate buffer (pH = 4.0). Rats with blood glucose levels ≥250 mg/dL at 48 h after STZ administration were considered diabetic. In this study, a commercially available liquid extract of Calendula officinalis (Immu-Nat, Immunat Herbal Medicine Inc., Bodrum, Türkiye) was used. The preparation was supplied as a 250-mL liquid herbal extract. According to the manufacturer's specifications, the extract contained 12 mg of total flavonoids, expressed as catechin equivalents (CE), and 37.1 mg of total phenolic compounds, expressed as gallic acid equivalents (GAE), per 5 mL. The reported antioxidant activity was expressed as an EC₅₀ value of 7.91 µL/mg DPPH. The liquid preparation was administered directly by oral gavage without prior dilution at a reported dose of 250 mg/kg for six weeks [26,27].

Collection of gingival tissue samples

At the end of the experimental period, general anesthesia was induced in all animals using a combination of ketamine (50 mg/kg) and xylazine (10 mg/kg), followed by sacrifice by exsanguination. A portion of the gingival tissue samples obtained by gingivectomy was stored at −80°C for biochemical analyses, while samples allocated for histological evaluation were fixed in 10% neutral buffered formalin.

Tissue homogenization and protein extraction

Frozen gingival tissues were thawed before analysis and placed in RIPA lysis buffer containing protease and phosphatase inhibitor cocktails. The tissues were homogenized using an AllSheng tissue homogenizer at 4350 rpm for 1 min. Following homogenization, the samples were centrifuged at +4°C for 20 min, and the resulting supernatants were transferred to new microcentrifuge tubes. Protein extracts were stored at −80°C until analysis.

Total protein determination

Total protein concentrations in the tissue extracts were determined using the Abbkine BCA Protein Assay Kit (Cat. No. KTD3001; Abbkine Scientific Co., Ltd., Wuhan, China) according to the manufacturer's protocol. Optical density (OD) measurements were performed at a wavelength of 562 nm using a microplate reader. Protein concentrations were calculated using a standard curve generated with bovine serum albumin.

ELISA analyses

The target protein levels were determined using specific ELISA kits for COX-2 (#YLA0104RA) and IL-1β (#YLA0030RA) (YLBiont, Shanghai YL Biotech Co., Ltd., Shanghai, China). All experimental procedures were performed step by step in accordance with the manufacturer's recommended protocols. At the end of the reactions, OD measurements were performed at 450 nm using a microplate reader. The resulting absorbance values were directly converted to concentration values using a four-parameter logistic regression model constructed from the absorbance values of the standard series. Finally, to normalize biological variation, the target protein amounts were normalized to the total protein concentrations determined by the BCA assay, and the results were standardized as the amount of the respective protein per 10 mg/mL of total protein.

Histopathological examination

Following completion of the experimental procedure, the teeth and associated gingival tissue samples were fixed in 10% neutral buffered formaldehyde. After fixation, the samples were placed in 10% formic acid solution for decalcification. Following decalcification, the samples underwent routine histological tissue processing using an automated tissue processor (Leica TP1020, Nussloch, Germany) and were embedded in paraffin. Serial sections of 5 µm thickness were obtained from the paraffin blocks using a microtome. The sections were stained with hematoxylin and eosin (H&E) for general histological evaluation and Masson's trichrome for evaluation of collagen fibers in the connective tissue. The slides were evaluated and imaged under a light microscope equipped with a Carl Zeiss Axiocam ERc5 digital camera system (Carl Zeiss Microscopy GmbH, Jena, Germany).

Statistical analysis

Statistical analyses were performed using IBM SPSS Statistics for Windows, Version 25 (Released 2017; IBM Corp., Armonk, New York, United States). The normality of the data distribution was assessed using the Shapiro-Wilk test. The nonparametric Mann-Whitney U test was used for biochemical comparisons between groups. COX-2 and IL-1β levels were separately compared between the control and *Calendula officinalis* groups, the C and DM groups, and the diabetes and *Calendula officinalis*-treated DM groups. These comparisons were selected to evaluate the effects of *Calendula officinalis* in non-diabetic animals, the biochemical changes associated with diabetes, and the effects of *Calendula officinalis* administration under diabetic conditions, respectively. Given the relatively small sample size (n = 7 per group), the statistical findings were interpreted cautiously, considering the limited statistical power and the possibility of type II errors. Data were presented as mean ± standard deviation (mean ± SD). A p-value <0.05 was considered statistically significant.

## Results

Biochemical findings

ELISA analyses showed no statistically significant differences in gingival tissue COX-2 or IL-1β levels between the C and *Calendula officinalis* groups (p=0.620 for both). Compared with the C group, the DM group showed statistically significant increases in both COX-2 and IL-1β levels (p=0.011 and p=0.017, respectively) (Table 1).

**Table 1:** Comparison of gingival tissue COX-2 and IL-1β levels among the control, Calendula officinalis, and diabetes mellitus groups Data are presented as mean ± standard deviation (mean ± SD). C: Control; CO: Calendula officinalis; DM: Diabetes mellitus; COX-2: Cyclooxygenase-2; IL-1β: interleukin-1β. Comparisons between groups were performed using the Mann–Whitney U test. *p<0.05 was considered statistically significant.

Parameter	C (mean ± SD)	CO (mean ± SD)	DM (mean ± SD)	U (C vs CO)	p-value (C vs CO)	U (C vs DM)	p-value (C vs DM)
COX-2 (ng/mL)	11.65 ± 4.80	11.08 ± 4.62	40.99 ± 22.67	20	0.62	5	0.011*
IL-1β (pg/mL)	3331.32 ± 1268.49	3360.67 ± 1195.24	12207.73 ± 6331.38	20	0.62	6	0.017*

In the DM group treated with *Calendula officinalis*, both COX-2 and IL-1β levels were significantly lower than those in the DM group (p=0.026 for both parameters) (Table 2).

**Table 2:** Comparison of gingival tissue COX-2 and IL-1β levels between the diabetes and Calendula officinalis-treated diabetes groups Data are presented as mean ± standard deviation (mean ± SD). CO: Calendula officinalis; DM: Diabetes mellitus; COX-2: Cyclooxygenase-2; IL-1β: interleukin-1β. Comparisons between groups were performed using the Mann–Whitney U test. *p<0.05 was considered statistically significant.

Parameter	DM (mean ± SD)	DM+CO (mean ± SD)	U	p-value
COX-2 (ng/mL)	40.99 ± 22.67	11.72 ± 0.76	7	0.026*
IL-1β (pg/mL)	12207.73 ± 6331.38	3640.44 ± 549.02	7	0.026*

Histopathological evaluation

In the C group, the overall histological organization of the gingival tissue was preserved. The keratinized stratified squamous epithelium exhibited a regular morphological appearance. The epithelial-connective tissue junction was distinct, and the epithelial projections showed a regular organization. The lamina propria consisted of dense irregular connective tissue, with densely arranged collagen fibers and identifiable connective tissue cells and small blood vessels. In sections from the DM group, although the overall integrity of the gingival tissue was preserved, several irregularities in epithelial and connective tissue organization were observed compared with the C group. Focal variations in the thickness of the keratinized stratified squamous epithelium, separation of the superficial keratin layer, and structural irregularities were noted. The lamina propria exhibited a relatively cell-poor appearance. In the DM group treated with *Calendula officinalis*, although focal separation of the keratin layer was observed, the epithelial-connective tissue junction appeared more regular and distinct than that in the DM group. Connective tissue cells in the lamina propria were more prominent, and the connective tissue exhibited a more regular organization (Figures 1, 2).

**Figure 1:**
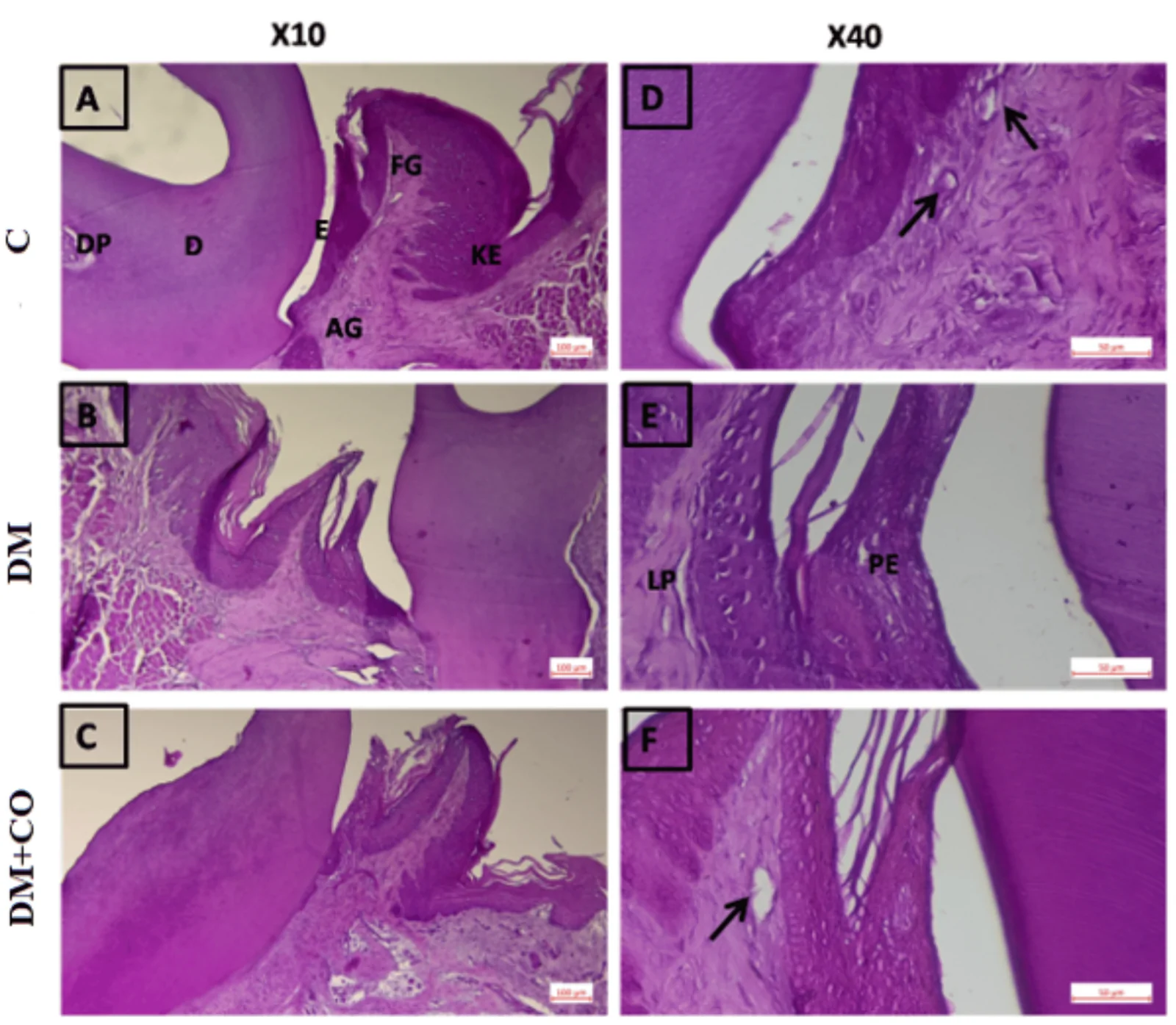
Representative light microscopy images of H&E-stained gingival tissue sections from the C, DM, and DM+CO groups. (A–C) Tooth and gingival tissues at ×10 magnification, showing dental pulp (DP), dentin (D), enamel space (E), free gingiva (FG), attached gingiva (AG), and keratinized epithelium (KE). (D–F) Middle region of the free gingiva at ×40 magnification, showing parakeratinized epithelium (PE), lamina propria (LP), and blood vessel (arrow). Scale bars: 100 μm for ×10 images and 50 μm for ×40 images C: Control; CO: Calendula officinalis; DM: Diabetes mellitus.

**Figure 2:**
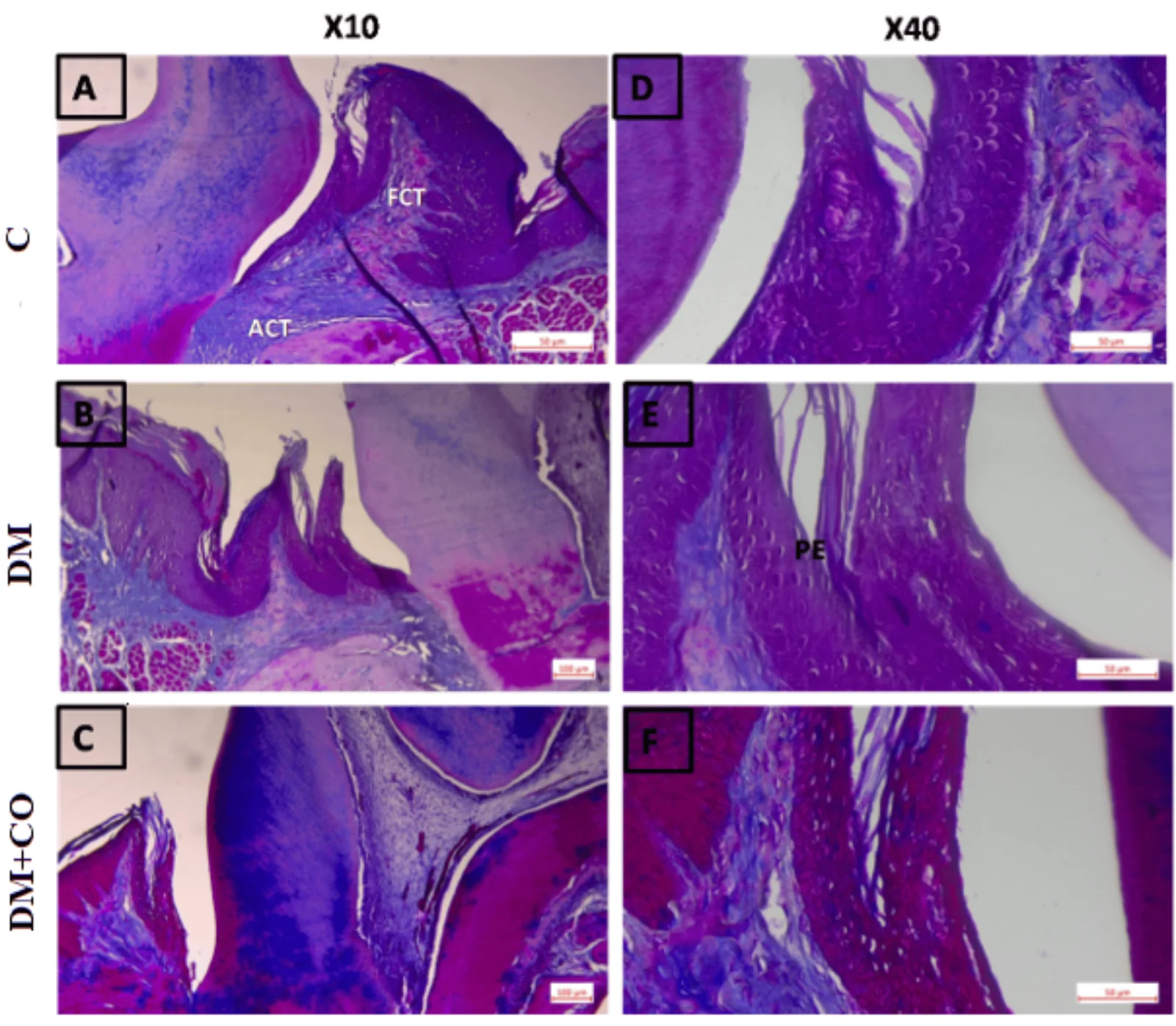
Representative light microscopy images of Masson's trichrome (MT)-stained gingival tissue sections from the C, DM, and DM+CO groups. (A–C) Gingival tissue at ×10 magnification, showing free gingival connective tissue (FCT) and attached gingival connective tissue (ACT). (D–F) Middle region of the free gingiva at ×40 magnification. Masson's trichrome staining. Scale bars: 100 μm for ×10 images and 50 μm for ×40 images C: Control; CO: Calendula officinalis; DM: Diabetes mellitus.

## Discussion

In this study, the effects of *Calendula officinalis* administration on inflammatory changes in gingival tissue were evaluated biochemically and histopathologically in a streptozotocin-induced experimental diabetes model. Gingival COX-2 and IL-1β levels were significantly increased in the DM group compared with the C group, whereas both markers were significantly lower in the DM group treated with *Calendula officinalis* than in the DM group. Histopathological evaluation revealed several epithelial and connective tissue irregularities in the DM group, whereas a more regular morphological appearance of the epithelial-connective tissue junction and connective tissue organization was observed in the DM group treated with *Calendula officinalis*. These findings suggest that *Calendula officinalis* administration may be associated with a reduction in the increased inflammatory response in gingival tissue under experimental diabetic conditions and that the observed histopathological changes accompanied these biochemical findings.

The hyperglycemic microenvironment that develops in diabetes leads to a series of metabolic and immunological alterations that enhance the inflammatory response and disrupt tissue homeostasis in periodontal tissues. Under high-glucose conditions, increased ROS production and AGE accumulation create a microenvironment in periodontal tissues in which oxidative stress and inflammation reinforce each other, while the interaction of AGEs with RAGE may activate the TLR/MyD88/NF-κB signaling pathway, thereby increasing the expression of proinflammatory mediators such as IL-1β and TNF-α and matrix metalloproteinases (MMPs) [28,29]. AGE accumulation also reduces type I and III collagen expression and impairs cell migration in gingival fibroblasts, while increased MMP activity induced by inflammatory mediators further promotes extracellular matrix (ECM) degradation [30,31]. The effects of hyperglycemia on immune cells also contribute to this process. Under diabetic conditions, despite impaired neutrophil chemotaxis and function, increased release of MMPs and ROS in periodontal tissues, together with the acquisition of a proinflammatory phenotype by macrophages and increased production of various inflammatory mediators, including IL-1β, contribute to the persistence of periodontal inflammation [32,33]. Macrophage pyroptosis and ROS-NLRP3 inflammasome activation associated with impaired autophagy under high-glucose conditions represent an additional mechanism through which the IL-1β-dominant inflammatory response may be enhanced [34,35]. Furthermore, high glucose disrupts the gingival epithelial barrier and increases the release of inflammatory mediators while suppressing collagen synthesis, proliferation, and migration in periodontal fibroblasts, further accentuating the effects of the diabetic inflammatory microenvironment on periodontal tissue integrity and repair capacity [36]. The ability of a high-glucose microenvironment to increase PGE2 production together with IL-1β in LPS-stimulated monocytes suggests that prostaglandin-mediated inflammatory mechanisms, in addition to cytokine responses, may contribute to periodontal inflammation associated with diabetes [37]. COX-2 is an important inducible enzyme that contributes to PGE2 synthesis during periodontal inflammation, and IL-1β and bacterial stimuli have been shown to enhance PGE2 production by increasing COX-2 expression in gingival fibroblasts [38].

Consistent with this mechanistic framework, gingival IL-1β and COX-2 levels were significantly increased in the DM group compared with the C group in the present study. This finding is consistent with previous experimental studies demonstrating an enhanced proinflammatory response in gingival and periodontal tissues under experimental diabetic conditions [29,39,40]. Similarly, Silva et al. [41], in their study evaluating periodontal inflammation in STZ-induced diabetic rats, demonstrated alterations in gingival COX-2 expression and reported that this pathway may play a role in diabetes-associated periodontal inflammation.

Traditional and complementary medicine approaches are widely used worldwide for therapeutic purposes [42]. *Calendula officinalis* has a broad phytochemical profile, including terpenoids, flavonoids, triterpenes, phenolic compounds, carotenoids, saponins, sterols, tocopherols, and various fatty acids, which contribute to its pharmacological effects. In particular, flavonoids such as quercetin, isorhamnetin, and isoquercetin, as well as carotenoids, triterpene alcohols, and their esterified derivatives, are among the major bioactive constituents of the plant [25]. The rich phytochemical composition of *Calendula officinalis* has also been associated with its anti-inflammatory properties. Various secondary metabolites, including alkaloids, tannins, flavonoids, essential oils, sterols, saponins, carotenoids, and triterpene alcohols, have been reported to contribute to this anti-inflammatory activity [43]. In particular, triterpenoid fatty acid esters found in Calendula flowers have been identified as important constituents responsible for the anti-inflammatory effect, with lauryl, myristoyl, and palmitoyl esters of faradiol being among the prominent compounds [22]. Experimental findings suggest that the anti-inflammatory effects of *Calendula officinalis* may be associated with the suppression of COX-2 and the production of proinflammatory cytokines such as IL-1β, IL-6, TNF-α, and IFN-γ [22,44]. In addition, its high flavonoid and phenolic compound content contributes to its antioxidant activity. These compounds have been reported to exhibit free radical-scavenging and chelating properties, and *Calendula officinalis* extracts have also demonstrated marked antioxidant activity [45,46].

The anti-inflammatory and antioxidant effects of *Calendula officinalis* are further supported by experimental studies conducted in periodontal tissues. Alexandre et al. [47] reported that *Calendula officinalis* administration reduced gingival IL-1β and TNF-α levels and MPO activity and limited alveolar bone loss in a ligature-induced experimental periodontitis model. Similarly, Lima et al. [48] demonstrated that *Calendula officinalis* administration reduced gingival oxidative stress, supported antioxidant defense components such as GSH, SOD, and CAT, decreased MDA levels, and contributed to the preservation of periodontal collagen fibers and alveolar bone. Furthermore, Tanideh et al. [49] reported favorable changes in antioxidant, inflammatory, and histopathological parameters in herbal treatment groups that included *Calendula officinalis*, with a marked reduction in IL-1β levels particularly in the *Calendula officinalis* and *Hypericum perforatum* combination group. Consistent with these findings, the significantly lower gingival IL-1β and COX-2 levels observed in *Calendula officinalis*-treated diabetic rats compared with untreated diabetic rats in the present study suggest that *Calendula officinalis* may modulate the increased gingival inflammatory response under diabetic conditions.

Structural deterioration in diabetic gingiva is characterized, in part, by alterations in the epithelial barrier. Narukawa et al. [50] demonstrated that chronic high-glucose conditions reduced the expression of intercellular junction proteins, including Claudin-1, ZO-1, and E-cadherin, in gingival epithelial cells. These changes were accompanied by thinning of the epithelial layer, irregular intercellular spaces, and increased epithelial permeability. AGE-RAGE signaling and increased oxidative stress have been reported to contribute to this barrier dysfunction through ERK1/2 activation [50]. In gingival connective tissue, MMPs are among the major regulators of ECM degradation, and disruption of the MMP/TIMP balance has been associated with an imbalance between matrix synthesis and degradation [51]. Kojima et al. [52] detected an SETD1A-mediated increase in H3K4me3 in the gingival tissue of STZ-induced diabetic rats and demonstrated increased MMP-1 and MMP-13 expression and MMP/TIMP ratios in human gingival fibroblasts exposed to high glucose. These findings indicate that hyperglycemia may shift the molecular balance in gingival fibroblasts toward matrix degradation [52].

Consistent with these mechanistic findings, histopathological evaluation in the present study revealed several morphological differences among the groups. In the DM group, alterations in gingival epithelial thickness and structural organization, separation of the superficial keratin layer, and a relatively cell-poor appearance of the lamina propria were observed. In contrast, in the DM group treated with *Calendula officinalis*, the epithelial-connective tissue junction appeared more regular and distinct, and the connective tissue exhibited a more regular organization. Previous experimental periodontitis models reporting reduced periodontal inflammation and tissue damage and better preservation of collagen fibers following *Calendula officinalis* administration support the histopathological observations of the present study [47,48]. Nevertheless, because the histopathological assessment in the present study was qualitative, the observed morphological differences should not be interpreted as evidence of a direct tissue-protective or regenerative effect of *Calendula officinalis*, but rather as histological observations accompanying the biochemical findings.

This study has several limitations. First, the use of seven animals per group resulted in a limited sample size and may have affected the statistical power. Second, the histopathological evaluation was qualitative, and parameters such as epithelial thickness, cellular density, and collagen organization were not assessed histomorphometrically. In addition, the inability to present histological images from the CO group limited the comparative assessment of the morphological effects of *Calendula officinalis* on non-diabetic gingival tissue. The inflammatory response was evaluated only on the basis of COX-2 and IL-1β levels; other proinflammatory cytokines and oxidative stress markers were not investigated. Furthermore, the lack of detailed phytochemical characterization and standardization of the *Calendula officinalis* extract limits the ability to associate the observed effects with specific bioactive constituents. The evaluation of *Calendula officinalis* at a single dose and for a single treatment duration also precluded assessment of dose-response relationships and time-dependent effects. Therefore, the findings should be interpreted with caution and supported by future studies with larger sample sizes, quantitative histomorphometric analyses, and multiple inflammatory and oxidative stress markers. Furthermore, as the findings were obtained from an experimental animal model, their applicability to human periodontal conditions remains uncertain, and further clinical studies are required to determine the potential therapeutic relevance of *Calendula officinalis* in humans.

## Conclusions

In this study, the effects of *Calendula officinalis* administration on the inflammatory response in gingival tissue were evaluated biochemically and histopathologically in a streptozotocin-induced experimental diabetes model. Gingival COX-2 and IL-1β levels were significantly increased in the DM group compared with the C group, whereas both markers were significantly decreased in the diabetic group treated with *Calendula officinalis* compared with the DM group. Histopathological examination also revealed epithelial and connective tissue irregularities in the diabetes group, whereas the epithelial-connective tissue junction and connective tissue organization exhibited a more regular morphological appearance in the *Calendula officinalis*-treated diabetic group. These findings suggest that *Calendula officinalis* may reduce the increased inflammatory response in gingival tissue under experimental diabetic conditions and that this effect may also be reflected at the histological level. Nevertheless, considering the limitations of the present study, these findings should be supported by further studies with larger sample sizes, quantitative histomorphometric analyses, and additional inflammatory and oxidative stress markers.
